# Distant Metastasis is the Dominant Cause of Treatment Failure after Lateral Lymph Node Dissection in Patients with Lateral Lymph Node Metastasis: Results of the Large Multicenter Lateral Node Study in China

**DOI:** 10.7150/jca.88009

**Published:** 2023-10-02

**Authors:** Kan Li, Feng Wang, Yujuan Jiang, Gong Hong, Zijin Li, Jianwei Liang, Weinan Wu, Wei Xing, Qian Liu

**Affiliations:** 1 Department of Surgery, Anyang Tumor Hospital, The Affiliated Anyang Tumor Hospital of Henan University of Science and Technology, Anyang, 455000, China.; 2 Department of Minimally Invasive Surgery, Beijing Chest Hospital, Capital Medical University, Beijing Tuberculosis and Thoracic Tumor Research Institute, Beijing, 101125, China.; 3 Department of Colorectal Surgery, National Cancer Center/National Clinical Research Center for Cancer/Cancer Hospital, Chinese Academy of Medical Sciences and Peking Union Medical College, Beijing, 100021, China.; 4 Department of General Surgery, Jilin City Central Hospital, Jilin, Jilin 132001, China; 5 Department of General Surgery, Hebei Province Hospital of Chinese Medicine, Affiliated Hospital of Hebei University of Chinese Medicine, Shijiazhuang, 050013, China.

**Keywords:** lateral lymph node metastasis, lateral lymph node dissection, distant metastasis, rectal cancer, preoperative chemotherapy, total neoadjuvant therapy.

## Abstract

**Background:** Lateral lymph node (LLN) metastases (LLNM) are often associated with poor prognosis. This study aimed to investigate the prognostic significance and postoperative recurrence pattern in rectal cancer patients with LLNM after LLN dissection (LLND).

**Materials and Methods:** This is a multicenter retrospective case-control study where propensity score-matched (PSM) analysis was introduced. From January 2012 to December 2019, 259 patients with clinical suspicion of LLNM who underwent LLND without neoadjuvant therapy were included in the study. They were divided into the negative (n = 197) and positive (n = 62) LLN groups. Primary endpoints were 3-year recurrence-free survival (RFS), local recurrence-free survival (LRFS), and distant metastasis-free survival (DMFS).

**Results:** After PSM, the DMFS rate in the positive LLN group was significantly worse (67.9 vs. 52.5%, *P* = 0.012). Pathological LLNM (HR, 3.07; 95% CI, 1.55-6.05; *P* = 0.001) were independent prognostic factors for DMFS. Patients in the positive LLN group had a higher proportion of distant metastases in all recurrence patterns (92.3% vs 82.6%). Among patients with LLN metastasis, metastases to the common iliac and external iliac arteries were the independent prognostic factor for DMFS (HR: 2.85; 95% CI, 1.31-4.67; *P* = 0.042). No significant different was observed for prognosis between patients with metastases to the obturator or internal iliac vessels and patients with a N2b stage.

**Conclusion:** Distant metastasis is the main cause of treatment failure after LLND in patients with LLNM. Because of the low completion rate of adjuvant chemotherapy, preoperative chemotherapy or total neoadjuvant therapy may be considered before LLND. In addition, patients with metastasis to external iliac and common iliac vessels have an extremely poor prognosis, and systemic chemotherapy instead of LLND should be recommended.

## Introduction

Developing countries show increasing trends in incidence and mortality of rectal cancer, especially in males and populations ≥50 years^1^, ^2^. Lateral lymph nodes (LLN) are one of the common lymphatic drainage routes in patients with middle-low rectal cancer, and conventional total mesorectal excision (TME) surgery cannot dissect LLN metastases (LLNM). The JCOG0212 trial has demonstrated that prophylactic lateral lymph node dissection (LLND) can be effective in suppressing the local recurrence in the lateral pelvic area, but only 7% of patients were pathologically confirmed to have positive LLN, so prophylactic LLND should not be promoted^3^.

In China, therapeutic LLND is usually performed only in patients with clinical suspicion of LLNM. However, the therapeutic effect and prognostic significance of LLND remain unclear. In addition, rectal cancer gradually tends to be treated with comprehensive therapy in recent years^4^, ^5^. It is necessary to optimize the current treatment strategy for LLN metastasis by exploring and analyzing the recurrence pattern of patients with LLNM after LLND. Therefore, we conducted a multicenter retrospective study using propensity score-matched (PSM) analysis to investigate the therapeutic effect and prognostic significance of LLND in patients with LPN metastasis. In addition, we combined the postoperative recurrence pattern and the location of LLN metastasis to optimize the treatment strategy of LLN metastasis and improve the value and significance of LLND.

## Method

### Patients

This was a multicenter retrospective case-control study based on a registry database. Clinical middle-low advanced rectal cancer (cT3-T4/cN+) patients with clinical suspicion of LLNM who underwent TME with LLND were included from three hospitals of the Chinese Lateral Node Collaborative Group from January 2012 to December 2019, including Cancer Hospital affiliated with the Chinese Academy of Medical Sciences, Peking University First Hospital, and Peking Union Medical College. The exclusion criteria were as follows: (1) patients with stage IV, (2) patients who underwent neoadjuvant therapy, (3) patients who underwent total pelvic exenteration, local resection, R2 resection, and (4) patients with history of other malignant tumors. The study design received ethical approval from each hospital and was registered (NCT04850027) at ClinicalTrials.gov. All enrolled patients signed informed consent, and all the procedures of the study were in accordance with the tenets of the Declaration of Helsinki.

### Diagnostic criteria

The status of LLN, such as short diameter, edge, shape, heterogeneity, and quantity, was assessed and determined by two radiologists based on magnetic resonance imaging (MRI). Clinical LLNM can be diagnosed by meeting any of the following diagnostic criteria: (1) ≥ 5 mm in short diameter, (2) malignant features (internal inhomogeneous, irregular borders and irregular shape) regardless of short diameter. TNM staging was performed using the American Joint Committee on Cancer (AJCC) staging system (8th edition)^6^, ^7^. The Clavien-Dindo classification system was used to grade the postoperative complications^8^.

### Treatment strategies

All patients were discussed in a multidisciplinary team meeting (MDT) that incorporated radiologists and medical and surgical oncologists to determine the treatment strategies, such as surgical approach, operative type, and adjuvant chemotherapy, for individual patients. According to National Comprehensive Cancer Network (NCCN) guidelines and European Society for Medical Oncology (EMSO), neoadjuvant chemoradiotherapy (nCRT) followed by TME without LLND is recommended for LLNM. In our center, selective LLND after nCRT were advised to patients with clinical evidence of LLNM. After considering the patient's financial situation and physical condition, the decision to administer nCRT or not was made by the multidisciplinary team. LLND was performed appropriately based on the location of enlarged LLN found by MRI. Bilateral LLND is not routinely performed and can only be used in patients with bilateral enlarged LLN detected on MRI. According to the Japanese Society for Cancer of the Colon and Rectum (JSCCR), the extent of LLND includes the common iliac vessel regions, the internal iliac vessel regions, the obturator region, and the external iliac vessel region. The dissected lymph nodes were classified according to the above areas and pathologically examined separately.

### Follow-up

Patients were scheduled for outpatient follow-up, with serum tumor markers (CEA and CA19-9) every 3 months, and a CT examination every 6 months in the first three years. Three years after the operation, the patients were scheduled for outpatient follow-up every 6 months. Relapse included local recurrence (LR) and distant metastases. For patients with LR, a pelvic MRI was performed to identify the location of recurrence. LR can be classified into central (anterior, presacral, anastomotic site, or perineal) and lateral pelvic regions. The endpoints of the present study were 3-year recurrence-free survival (RFS), 3-year local recurrence-free survival (LRFS), and 3-year distant metastasis-free survival (DMFS).

### Statistical analysis

Data analysis was conducted using SPSS for Windows (version 24.0; SPSS, Chicago, IL, USA). PSM was performed using logistic regression to reduce the imbalance between the two groups. The matching ratio was 1:1 and the covariates included age, sex, body mass index (BMI), American Society of Anesthesiologists (ASA) category, distance from anal verge, carcinoembryonic antigen (CEA) level, surgical approach, histology, pT stage, pN stage, tumor size, perineural invasion, and lymphatic invasion.

Continuous and categorical variables were compared using the t-test and chi-square test, respectively. The Kaplan-Meier method was used to calculate the RFS, LRFS, and DMFS; survival differences are compared using the log-rank test. Multivariate COX regression analysis was performed using co-variables with a relatively significant effect (*P* < 0.20) in the univariate analysis, and the effect of each variable was evaluated using the hazard ratio (HR) and 95% confidence interval (95% CI). *P* < 0.05 was defined as statistical significance.

## Results

### Details of patients

**Figure 1:**
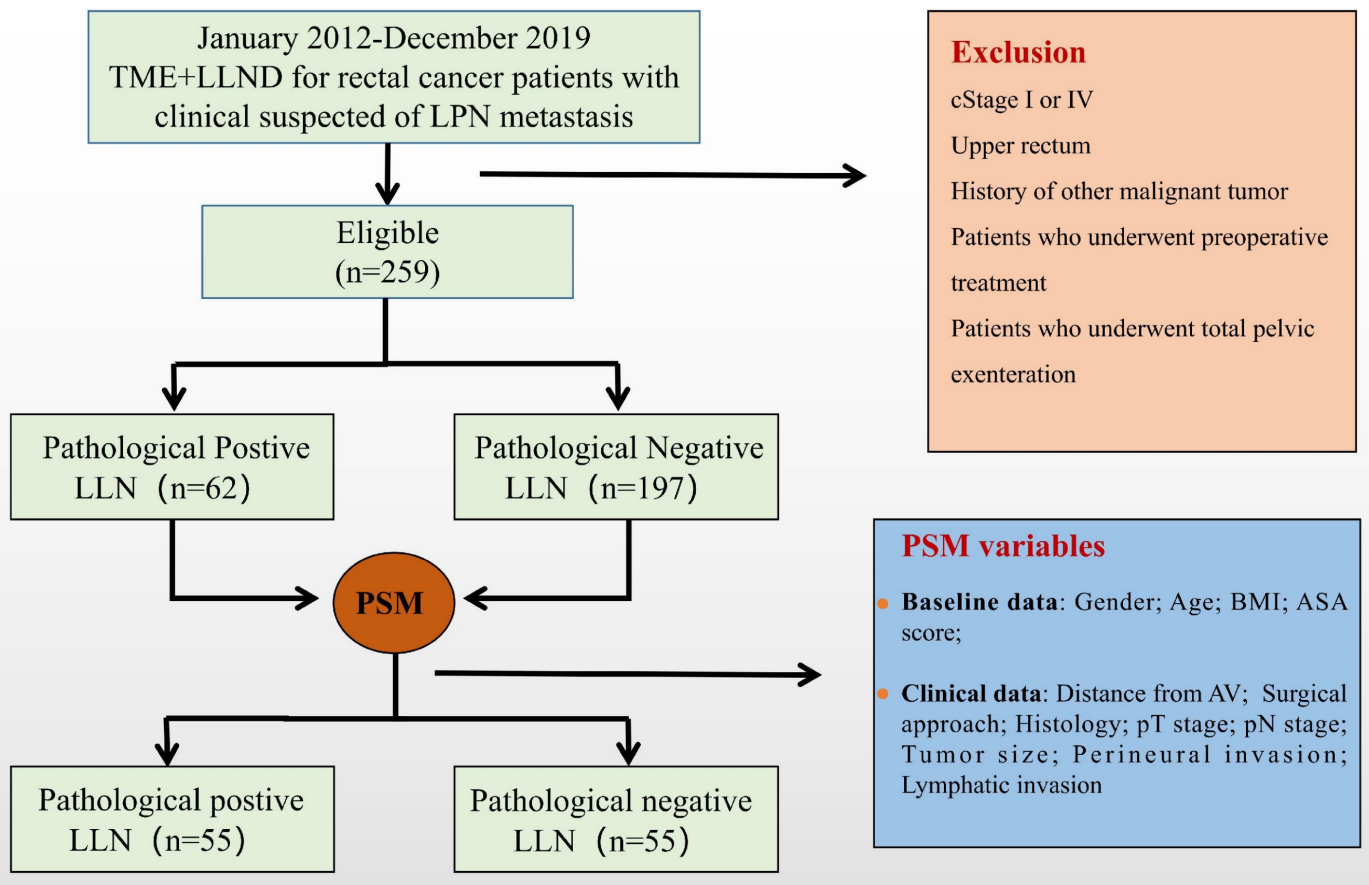
Research flowchart. TME, total mesorectal excision; LLN, lateral lymph node; LLND, lateral lymph node dissection; LLNM, lateral lymph node metastases; BMI, body mass index; ASA, American Society of Anesthesiologists, AV, anal verge

A total of 259 patients were included and divided into positive (n = 62) and negative (n = 197) LLN groups based on the pathological results. All enrolled patients were included in the PSM process to balance the variables between the two groups, and 55 matched pairs were selected (**Figure 1**). After matching, the positive and negative LLN groups were well-balanced in terms of clinical characteristics, pathological features, and perioperative variables (**Table 1 and Table 2**).

### Prognostic factors of LLND and postoperative recurrence pattern

**Figure 2:**
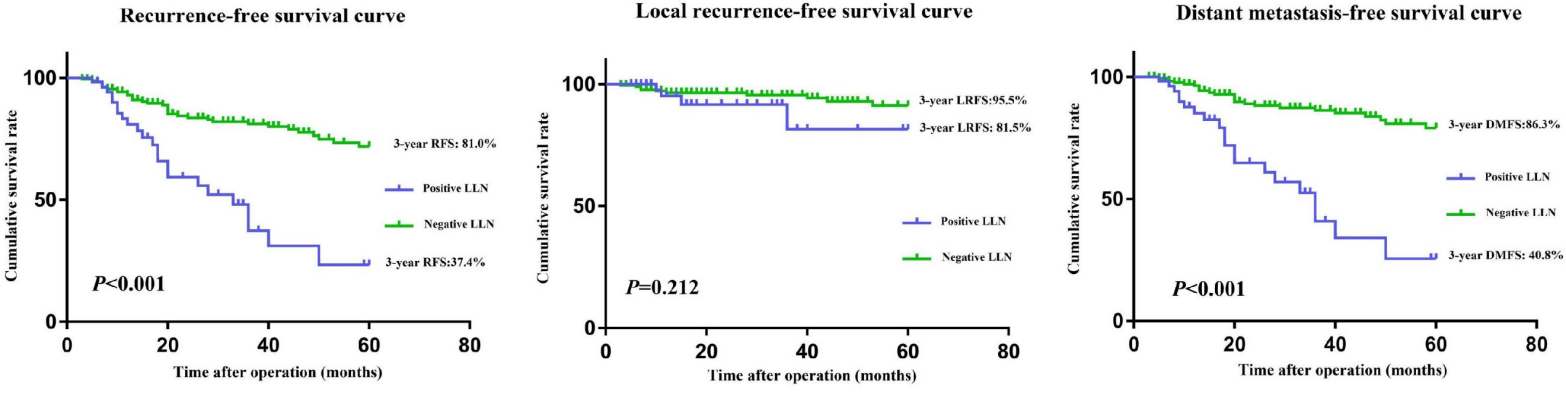
RFS **(A)**, LRFS **(B)**, and DMFS **(C)** curves of patients in positive and negative LLN groups before matching. RFS, recurrence-free survival; LRFS, local recurrence-free survival; DMFS, distant metastasis-free survival; LLN, lateral lymph node.

**Figure 3:**
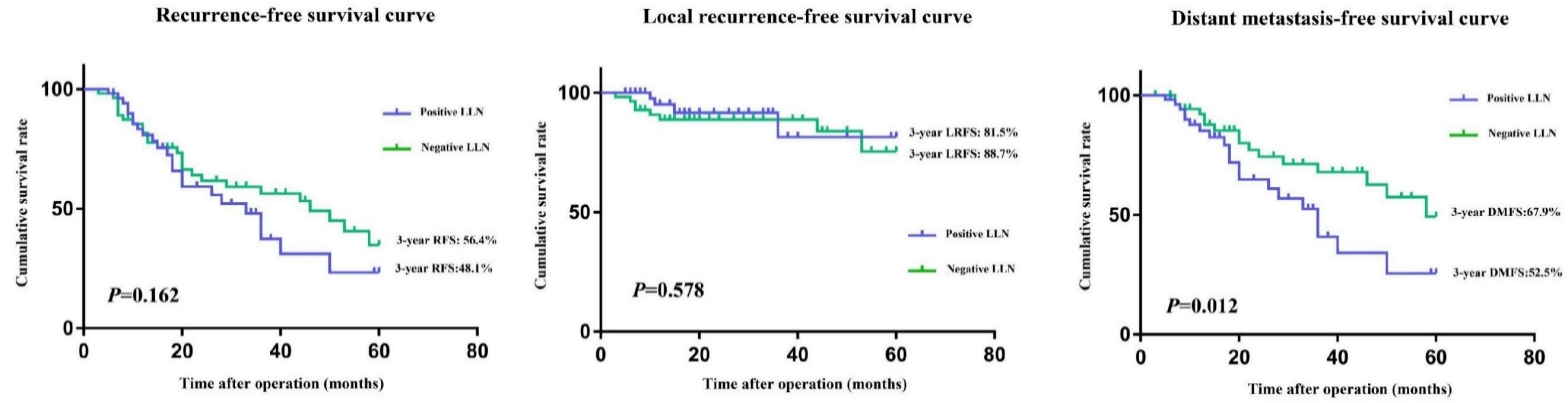
RFS **(A)**, LRFS **(B)**, and DMFS **(C)** curves of patients in positive and negative LLN groups after matching. RFS, recurrence-free survival; LRFS, local recurrence-free survival; DMFS, distant metastasis-free survival; LLN, lateral lymph node

The mean follow-up period for this study was 38.0 months; in this period, 58/259 patients had LR or distant metastasis. Before PSM, the 3-year RFS rate (81.0 vs. 37.4%, *P* <0.001) and 3-year DMFS rate (86.3 vs. 40.8%, *P* < 0.001) were significantly worse in the positive LLN group than that of the negative LLN group. However, the 3-year LRFS rates (95.5% vs 81.5%, *P*=0.212) were similar in both groups (**Figure 2A-C**). Although both groups had similar 3-year RFS rates (56.4% vs 48.1%, *P*=0.162) and 3-year LRFS rates (81.5 vs. 88.7%, *P* = 0.578) after matching, the 3-year DMFS rate (67.9 vs. 52.5%, *P* = 0.012) of patients in the positive LLN group, in comparison to that of the negative LLN group, were still significantly worse (**Figure 3A-C**).

Univariate and multivariate regression analyses of 259 patients with clinical suspicion of LLNM who underwent TME+LLND are shown in **Table 3**. The predictors of the LRFS were lymphatic invasion, pT stage, and pN stage (*P* < 0.02). In addition, the DMFS was associated with the CEA level, LPND procedure, histology, pN stage, and pathological LLNM (*P* < 0.02). Multivariate analysis revealed that lymphatic invasion (HR, 3.22; 95% CI, 1.52-8.91; *P* = 0.041) was an independent prognostic factor for LRFS; N2 stage (HR, 3.46; 95% CI, 1.49-8.03; *P* = 0.004) and pathological LLNM (HR, 3.07; 95% CI, 1.55-6.05; *P* = 0.001) were independent prognostic factors for DMFS. Supplementary Table 1 shows the regression analyses of 55 paired patients.

**Figure 4:**
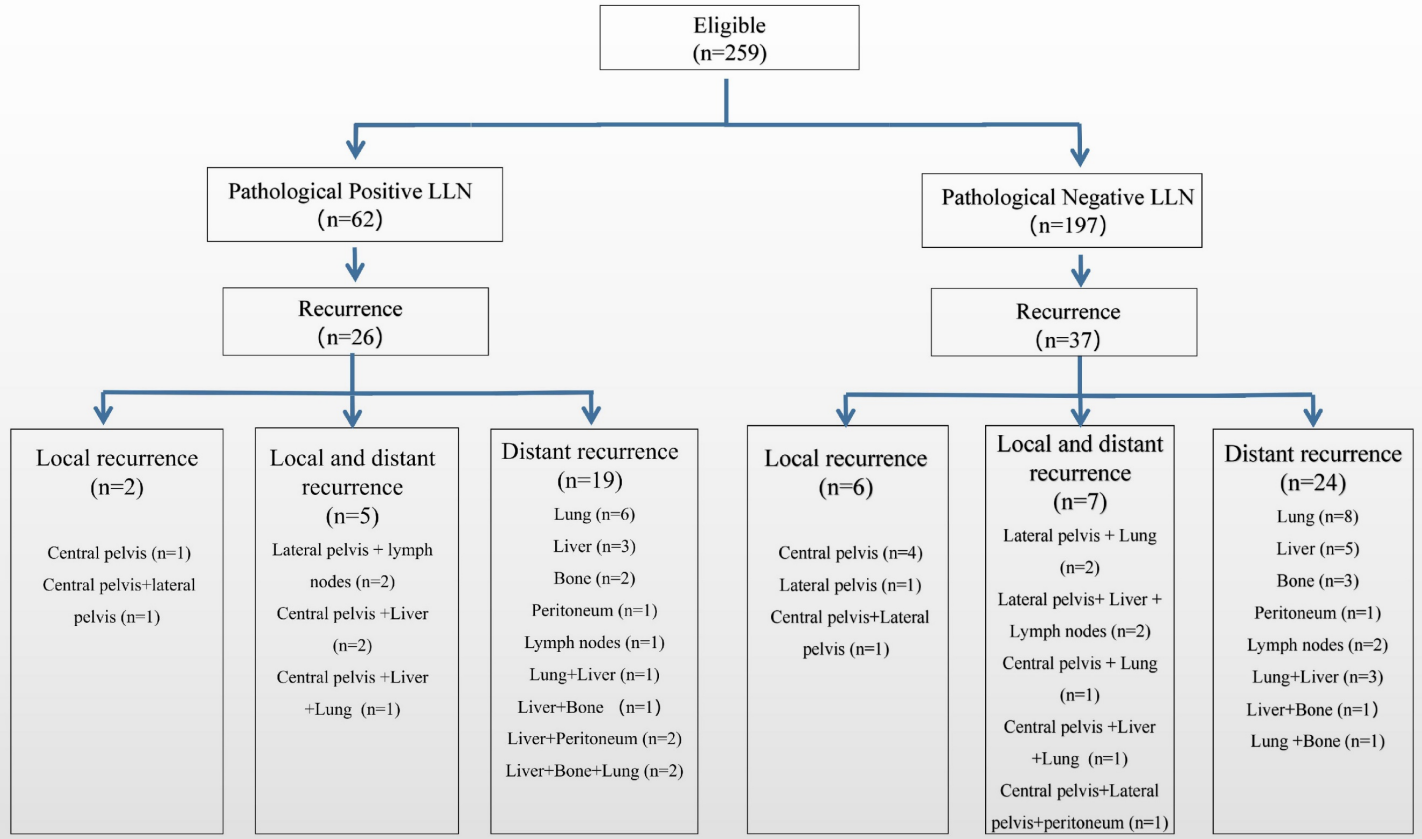
Recurrence rate of local and distant metastasis before matching. LLN, lateral lymph node; PSM, propensity score-matched.

**Figure 5:**
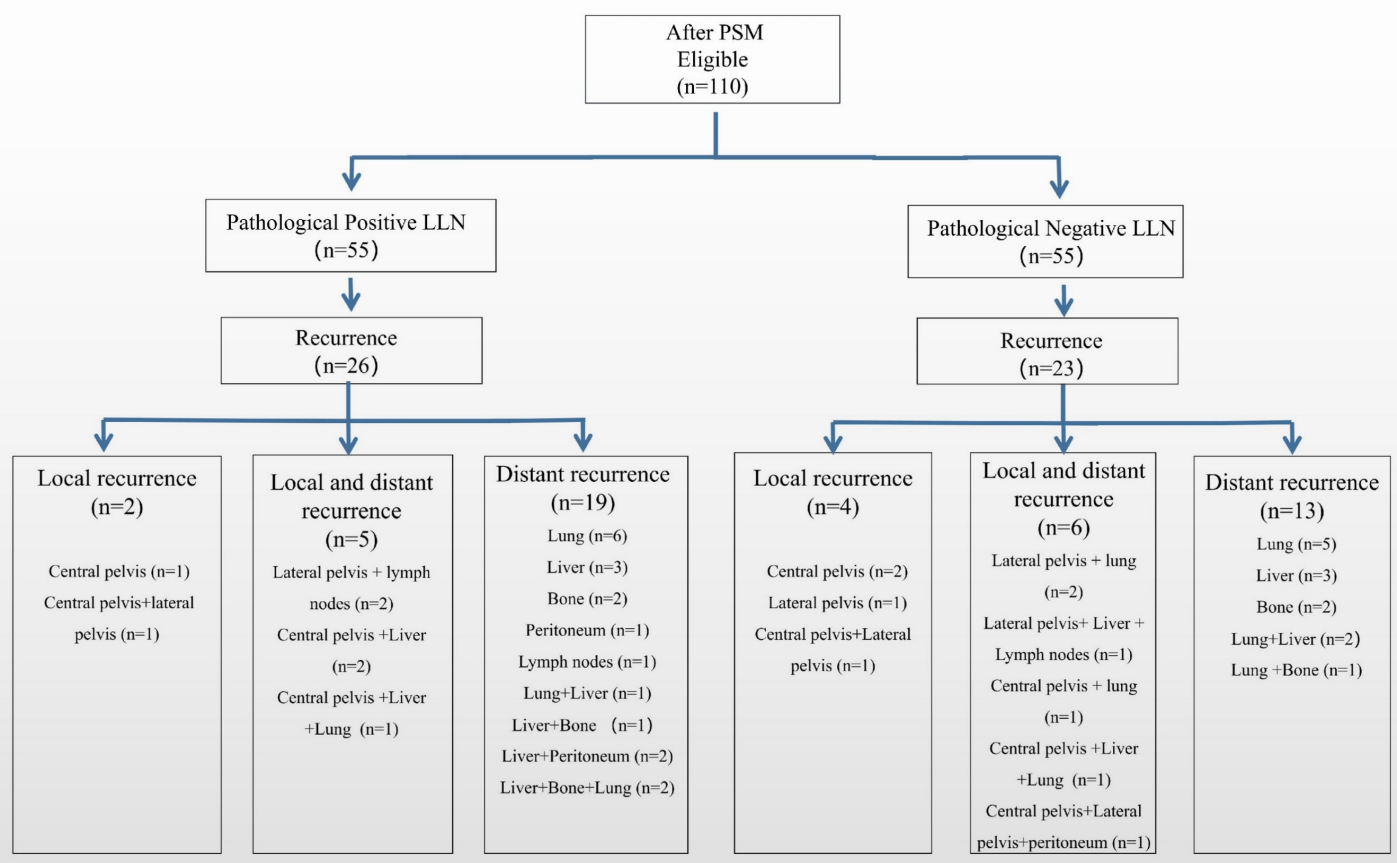
Recurrence rate of local and distant metastasis after matching. LLN, lateral lymph node; PSM, propensity score-matched.

**Figures 4 and 5** show a flowchart of recurrence up to 3 years after the surgery in both groups before and after matching. Before matching, 26/62 patients (41.9%) with positive LLN relapsed, seven (11.3%) experienced LR, and 24 (38.7%) had distant recurrence. Further, 37/197 (18.8%) patients with negative LLN relapsed: 13 (6.6%) experienced LR and 31 (15.7%) had distant recurrences. Patients in the positive LLN group had a higher proportion of distant metastases in all recurrence patterns than in the negative LLN group (92.3% vs. 83.8%). After matching, patients in the positive LLN group still had a higher proportion of distant metastases in all recurrence patterns than in the negative LLN group (92.3% vs 82.6%).

### Prognostic factors of DMFS in patients with LLNM

The univariate and multivariate regression analyses of DMFS in the 62 patients with pathological LLNM are shown in **Table 4**. Univariate analysis identified the LLNM locations, distance from the anal verge, and operative type as the predictors of OS (*P* < 0.20). Multivariate analysis revealed that the LLN metastasis to the common iliac and external iliac vessels was an independent prognostic factor for DMFS (HR: 2.85; 95% CI, 1.31-4.67; *P* = 0.042).

**Figure 6:**
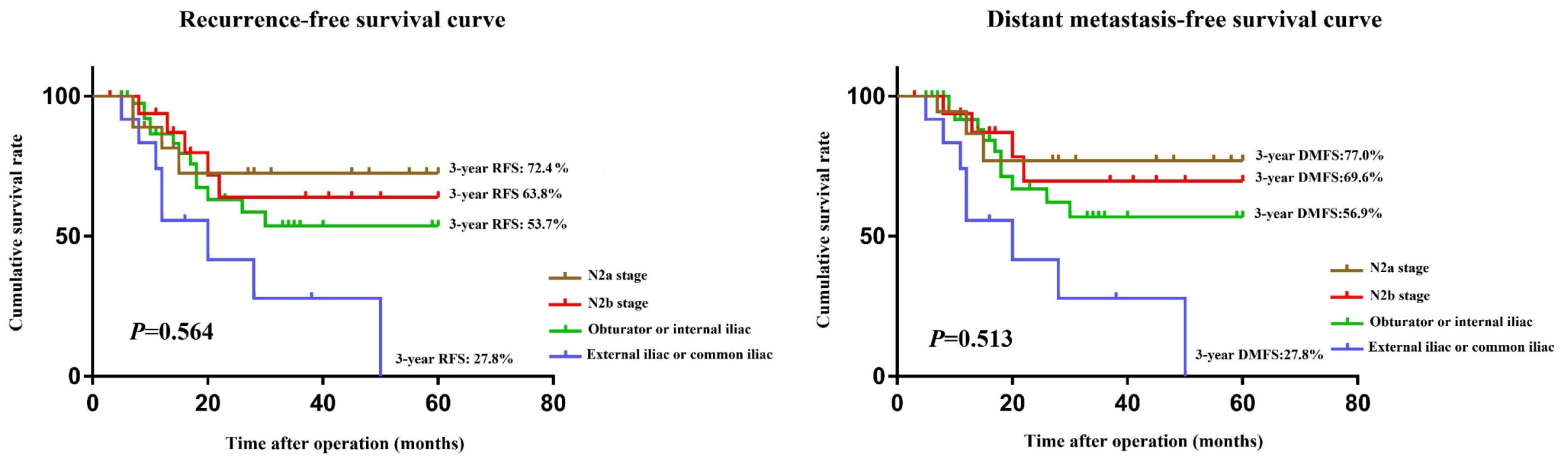
RFS **(A)** and DMFS **(B)** curves of patients with LLN metastasis and N2 stage after subgroup analysis. RFS, recurrence-free survival; DMFS, distant metastasis-free survival.

To further explore the survival outcomes of the location of the LLNM, we selected 36 patients with N2 stage from the negative LLN group and subdivided them into N2a stage (4-6 regional lymph node metastases) and N2b stage (≥7 regional lymph node metastases) according to the AJCC tumor staging system. The 3-year RFS (and the 3-year DMFS) rates of the N2a stage, N2b stage, LLN metastasis to the obturator or internal iliac, and LLN metastasis to the common iliac and external iliac vessels were 72.4% (77.0%), 63.8% (69.6%), 53.7% (56.9%), and 27.8% (27.8%), respectively (**Figure 6A, B**). The RFS (*P* = 0.564) and DMFS (*P* = 0.513) were not significantly different between patients with the LLN metastasis to the obturator or internal iliac vessels and N2b stage.

## Discussion

The standard of care for LLNM is different between Japan and Western countries. In Japan, LLNM is treated as a local metastasis with a focus on systemic lymph nodes dissection, i.e., TME plus LLND^9^; Europe and the United States regard it as such distant metastases, nCRT plus TME were performed, not routine LLND^10^. The optimal treatment strategy has not yet been developed in Eastern and Western countries. Numerous studies conducted recently have emphasized the value of preoperative neoadjuvant chemoradiotherapy in the combination therapy for LLNM^11^. Akiyoshi^12^, advocate preventive LLND, compared the survival outcomes of LLNM treated by LLND with or without nCRT and found that nCRT significantly improved the prognosis of LLNM compared with surgery alone. Currently, the combination of nCRT and LLND are considered as a mainstream treatment option for LLNM patients. A number of nations, including China, suggest selective LLND after neoadjuvant chemoradiotherapy (nCRT) for patients with clinical evidence of LLNM^13^.

There are various therapeutic strategies to treat LLNM, including bilateral LLND^14^, selective LLND only for swollen lymph detected on preoperative MRI^15^, and omission of LLND and replacement with neoadjuvant therapy^16^. Our previous study investigated the therapeutic benefits and effective range of LLND in patients with LLNM^17^, but that study included cases treated with neoadjuvant therapy, which often alters the status of lymph nodes, confounding the process of grouping based on pathological findings. Therefore, we conducted a multicenter retrospective case-control study to evaluate the therapeutic effect and prognostic significance of LLND alone in patients with LLN metastasis, and to explore the recurrence pattern after LLND to optimize the treatment strategy for LLN metastasis. This study demonstrated that after matching, although the LRFS of patients with LLNM after LLND was similar to that of patients without LLNM, DMFS was significantly lower, and distant metastasis was the main recurrence pattern for patients with LLNM. In addition, patients with metastasis limited to the internal iliac and obturator region can achieve a similar prognosis after LLND to those of patients with stage N2b stage, but patients with metastasis to external iliac and common iliac vessels have an extremely poor prognosis and may not benefit from LLND.

The rectum below the peritoneal reflection has three lymphatic drainage pathways: upper, lateral, and lower. When the upward lymphatic drainage pathway is blocked, tumor cells tend to metastasize to the lateral lymphatic drainage pathway. Obstruction of upper lymphatic drainage is usually due to excessive proliferation of tumor cells, so patients with LLNM often present with mesangial lymph node metastases^18^. Therefore, patients with lateral lymph node metastasis have a wider lymphatic metastasis area, a greater tumor burden, and are more likely to experience distant metastasis through the hemorrhagic metastasis pathway. In the present study, after balancing the relevant variables, we found that although patients with positive LLN could achieve similar local control (81.5 vs. 88.7%, *P* = 0.578) after LLND compared with patients with negative LLN, the 3-year DMFS rate (67.9 vs. 52.5%, *P* = 0.012) was still poor. In addition, distant metastasis is the most common recurrence pattern in patients with positive LLN.

Whether LLNM is a systemic or local disease determines the value and significance of LLND, but this issue has always been controversial in both Eastern and Western countries. In recent years, several studies have demonstrated that the location of LLNM is a crucial factor that affects the efficacy of LLND in patients with LLNM.^14^, ^19^-^22^ The 8th edition of the AJCC staging system has defined internal iliac lymph node metastases as regional diseases^7^. In addition, results from high-volume centers in Japan revealed that patients with metastasis confined to internal iliac and obturator areas could achieve similar survival outcomes after LLND to those with metastasis to the superior rectal artery area^14^. Similarly, Akiyoshi et al. conducted a Japanese Nationwide Multi-Institutional Study on Lateral Pelvic Lymph Node Metastasis, and the results showed that patients with metastases confined to the internal iliac lymph nodes have a prognosis similar to that of patients with N2a stage, and patients with metastases beyond the internal iliac nodes have a prognosis similar to that of patients with N2b stage^19^. In this study, the prognosis of patients with metastasis confined to the internal iliac nodes and obturator region were significantly better than those of patients with metastasis to external iliac and common iliac lymph node, and the 3-year RFS (53.7% vs 63%, *P* = 0.564) and 3-year DMFS (56.9% vs 69.6%, *P* = 0.513) of the former was similar to that of patients with N2b stage. Unlike the study by Akiyoshi et al., the present study included patients with metastases to both internal iliac and obturator regions for analysis, which led to differences in survival outcomes between groups. The survival benefit of LLND in patients with obturator lymph node metastases remains controversial. Chinese surgeons currently regard the internal iliac and obturator lymph nodes as regional lymph nodes, and patients with metastasis to these areas can benefit from LLND^23^, ^24^.

In recent years, the concept of multidisciplinary comprehensive treatment has played a positive role in improving the prognosis of LLNM from rectal cancer. Selective LLND after nCRT is currently the most common treatment strategy for rectal patients with LLNM^25^. In this study, the postoperative recurrence pattern was explored and it was found that patients in the positive LLN group had a higher proportion of distant metastases in all recurrence patterns than those in the negative LLN group (92.3% vs. 82.6%). However, relevant literature demonstrated that nCRT can improve local control but is less effective in reducing the risk of systemic metastasis for rectal cancer patients^26^. Since distant metastasis is the dominant recurrence type in patients with LLNM, enhanced systemic chemotherapy is an effective method to eliminate potential micro-metastases. However, poor compliance and severe complications may hinder the progression of adjuvant chemotherapy. In this study, the proportion of patients with LLNM who completed the full cycle of adjuvant chemotherapy after surgery was only 71.0%, and nearly 1/3 of the patients gave up chemotherapy for various reasons. Therefore, total neoadjuvant therapy, as a new paradigm for rectal cancer treatment, may be considered to improve therapeutic efficacy^27^, ^28^. In addition, we are currently conducting a phase III clinical study to explore the feasibility of replacing nCRT with three-drug-based preoperative chemotherapy in patients with LLNM, so as to ensure the downstaging effect and maximize the elimination of micro-metastases.

MRI is currently regarded as an accurate imaging modality in the preoperative evaluation of the nodal status^29^. The factors of lateral pelvic lymph nodes evaluated were short diameter, shape, border, and internal structure^30^. In this study, we used the short axis greater than 5mm as the criterion for judging suspected lymph node metastasis. It is known that about ≥50% of the involved nodes in rectal cancer are less than 5 mm in size, the size of lymph nodes in rectal cancer does not correspond with the presence or absence of metastases in lymph nodes^31^. This shows that using lymph node size alone as a criterion for assessing node-positivity is insufficient. And Kim et al^29^ reported even in patients who had only small lymph nodes less than 5 mm in diameter, the indistinct margin was helpful to predict lymph node positivity. Despite their small size, they also show desmoplastic reactions with/without perinodal tumor extension. Therefore, in order to prevent the missed suspected cases, we included patients with lymph node malignant features (internal inhomogeneous, irregular borders, and irregular shape) regardless of lymph node size. Previous literature has reported that the positive predictive value for the diagnosis of LLNM by MRI is 28.6%-51.6%^32^-^35^. Different diagnostic criteria for LLNM between institutions leads to large differences in reported positive predictive values. In this study, the diagnostic criteria for LLNM were too broad, and the positive rate was only 23.9% (62/259), which led to excessive and unnecessary LLND. In recent years, we have optimized and improved the diagnostic criteria for LLNM, and we suggested that patients with LLN short diameter ≥ 7mm after nCRT and adverse histological type can be diagnosed as LLNM, and such patients should be treated with LLND. According to the optimized diagnostic criteria for LLNM, the positive predictive value increased to > 50%^11^.

There are several limitations in present study. First, besides the relatively small sample size, retrospective multicenter studies have inherent selection bias and heterogeneity in treatment measure. However, selection bias was reduced by PSM using logistic regression. Second, the mean follow-up time was only 38.0 months, which is limited for adequately evaluating the 5-year RFS and DMFS.

## Conclusion

Distant metastasis is the main cause of treatment failure after LLND in patients with LLNM. Because of the low completion rate of adjuvant chemotherapy, preoperative chemotherapy or total neoadjuvant therapy may be considered before LLND. After LLND, patients with LLNM confined to the internal iliac and obturator regions appear to achieve comparable prognosis to those with N2b stage. However, patients with metastasis to external iliac and common iliac vessels have an extremely poor prognosis, and systemic chemotherapy instead of LLND should be recommended.

## Supplementary Material

Supplementary table.

## Figures and Tables

**Table 1:** The clinical and pathological characteristics before and after matching.

Variables	Original cohort			Matched cohort		
	Positive LLN (n=62)	Negative LLN (n=197)	*P*	Positive LLN (n=55)	Negative LLN (n=55)	*P*
Age (years, mean±SD)	57.9 ± 12.4	57.8 ± 11.2	0.900	56.6 ± 12.4	55.6 ± 13.0	0.686
Sex			0.096			0.565
Male	32 (51.6)	125 (63.5)		29 (52.7)	32 (58.2)	
Female	30 (48.4)	72 (36.5)		26 (47.3)	23 (41.8)	
BMI (kg/m^2^, mean±SD)	23.4 ± 2.7	24.1 ± 3.3	0.130	23.7 ± 2.8	24.0 ± 3.2	0.322
ASA category			0.632			1.000
I-II	60 (96.8)	193 (97.8)		55 (100.0)	54 (98.2)	
III	2 (3.2)	4 (2.2)		0 (0)	1 (1.8)	
Distance from anal verge (cm, mean ± SD)	4.5 ± 2.4	5.0 ± 2.5	0.184	4.5 ± 2.4	4.8 ± 2.8	0.566
CEA level(ng/ml)			0.016			0.445
≥5	31 (50.0)	65 (33.0)		28 (50.9)	24 (43.6)	
<5	31 (50.0)	132 (67.0)		27 (49.1)	31 (56.4)	
Surgical approach			0.133			0.539
Open	18 (29.0)	78 (39.6)		16 (29.1)	19 (34.5)	
Laparoscopic	44 (71.0)	119 (60.4)		39 (70.9)	36 (65.5)	
Histology			0.003			0.699
Moderate	30 (48.4)	136 (69.0)		33 (60.0)	31 (56.4)	
Poor/Mucinous/signet	32 (51.6)	61 (31.)		22 (40.0)	24 (43.6)	
pT stage			<0.001			1.000
T_1_ -T_2_	5 (8.1)	88 (44.7)		4 (7.3)	5 (9.1)	
T_3_-T_4_	57 (91.9)	109 (55.3)		51 (92.7)	50 (90.9)	
pN stage (mesorectal LN)			<0.001			0.957
N_0_	8 (12.9)	107 (54.3)		7 (12.7)	8 (14.5)	
N_1_	28 (45.2)	54 (27.4)		24 (43.6)	23 (41.8)	
N_2_	26 (41.9)	36 (18.3)		24 (43.6)	24 (43.6)	
Tumor size (cm, mean±SD)	4.6 ± 1.7	4.6 ± 2.0	0.988	4.5 ± 1.7	4.6 ± 1.6	0.881
Perineural invasion	40 (64.5)	88 (44.7)	0.006	39 (70.9)	34 (61.8)	0.313
Lymphatic invasion	36 (58.1)	80 (40.6)	0.016	31 (56.4)	29 (52.7)	0.702

Note: *LLN*, lateral lymph node; *BMI*, body mass index; *ASA*, American Society of Anesthesiologists; *CEA*, carcinoembryonic antigen.

**Table 2:** Operative and perioperative data before and after matching.

Variables	Original cohort			Matched cohort		
	Positive LLN (n=62)	Negative LLN (n=197)	*P*	Positive LLN (n=55)	Negative LLN (n=55)	*P*
Type of operation			0.119			0.840
Low anterior resection	32 (51.6)	125 (63.5)		29 (52.7)	32 (58.2)	
Abdominoperineal resection	27 (43.5)	69 (35.0)		24 (43.6)	21 (38.2)	
Hartmann procedure	3 (4.9)	3 (1.5)		2 (3.7)	2 (3.6)	
LLND procedure			0.168			0.279
Unilateral	47 (75.8)	131 (66.5)		43 (78.2)	38 (69.1)	
Bilateral	15 (24.2)	66 (33.5)		12 (21.8)	17 (30.9)	
Operative time, median (range) min	272 (140-742)	254 (125-600)	0.194	263 (140-742)	242 (135-550)	0.465
Estimated blood loss, median (range) ml	100 (10-200)	100 (10-500)	0.569	80 (10-200)	100 (20-300)	0.633
Postoperative complications (Grade≥2)	10 (16.1)	29 (14.7)	0.787	8 (14.5)	11 (20.0)	0.449
Anastomotic leakage	2 (3.2)	7 (3.6)	1.000	1 (1.8)	2 (3.6)	1.000
Ileus	4 (6.5)	4 (2.0)	0.182	3 (5.5)	2 (3.6)	1.000
Gastrointestinal hemorrhage	0 (0)	1 (0.5)	1.000	0 (0)	0 (0)	-
Urinary infection	1 (1.6)	5 (2.5)	1.000	1 (1.8)	1 (1.8)	1.000
Urinary retention	6 (9.7)	12 (6.1)	0.495	4 (7.3)	6 (10.9)	0.507
Renal failure	0 (0)	1 (0.5)	1.000	0 (0)	0 (0)	-
Pneumonia	1 (1.6)	5 (2.5)	1.000	1 (1.8)	1 (1.8)	1.000
Arrhythmia	0 (0)	2 (1.0)	1.000	0 (0)	0 (0)	-
Chylous ascites	0 (0)	3 (1.5)	1.000	0 (0)	2 (3.6)	0.495
Abdominal abscess	4 (6.5)	6 (3.0)	0.403	3 (5.5)	2 (3.6)	1.000
Abdominal or perineal incision infection	0 (0)	3 (1.5)	1.000	0 (0)	1 (1.8)	1.000
Neuropathy in lower limb	2 (3.2)	2 (1.0)	0.243	1 (1.8)	1 (1.8)	1.000
Mortality	1 (1.6)	0 (0)	1.000	0 (0)	0 (0)	-
Postoperative hospital stay, median (range) days	11 (5-52)	12 (4-67)	0.669	10 (5-52)	10 (4-64)	0.649
Completed adjuvant therapy	44 (71.0)	71 (36.0)	<0.001	40 (72.7)	35 (63.6)	0.306

**Table 3:** Univariate and multivariate regression analyses of 259 patients with clinical LLNM who underwent TME+LLND.

Variables	LRFS				DMFS			
	Univariate analysis		Multivariate analysis		Univariate analysis		Multivariate analysis	
	HR (95%CI)	*P*	HR (95%CI)	*P*	HR (95%CI)	*P*	HR (95%CI)	*P*
Sex: male/female	0.77 (0.27-2.22)	0.629			1.12 (0.61-2.06)	0.707		
Age at operation (≥65/<65years)	0.44 (0.27-5.70)	0.293			0.69 (0.32-1.48)	0.342		
CEA level (>5/≤5 ng/L)	1.92 (0.64-5.75)	0.242			1.76 (0.95-3.26)	0.074	1.31 (0.69-2.47)	0.406
Distance from anal verge (>5/≤5 cm)	0.90 (0.30-2.72)	0.857			0.67 (0.35-1.27)	0.219		
Operative type: laparoscopic/open	1.33 (0.44-4.01)	0.608			1.27 (0.69-2.35)	0.447		
LPND (Bilateral/Unilateral)	0.70 (0.23-2.11)	0.519			0.58 (0.30-1.10)	0.093	0.67 (0.34-1.33)	0.255
Histology (Poor, Mucinous or signet/moderate)	1.18 (0.21-6.55)	0.847			3.37 (1.12-10.11)	0.030	2.72 (0.86-8.43)	0.225
Lymphatic invasion (yes/no)	1.41 (0.28-7.00)	0.674			0.92 (0.27-3.14)	0.890		
Perineural invasion (yes/no)	3.78 (1.42-8.42)	0.033	3.22 (1.52-8.91)	0.041	2.01 (0.61-6.66)	0.254		
pT stage (T3-T4/T1-T2)	2.68 (0.74-9.67)	0.133	4.42 (0.76-11.42)	0.251	0.73 (0.23-2.36)	0.598		
pN stage (mesorectal LN)								
N0	-	-	-	-	-	-	-	-
N1	2.75 (1.62-9.78)	0.042	2.21 (0.91-9.75)	0.142	2.30 (1.03-5.15)	0.042	1.96 (0.82-4.71)	0.132
N2	3.30 (2.35-9.46)	0.004	3.65 (0.95-8.86)	0.084	4.64 (2.21-9.71)	<0.001	3.46 (1.49-8.03)	0.004
Pathological LLNM (yes/no)	2.10 (0.64-6.92)	0.222			4.88 (2.65-8.97)	<0.001	3.07 (1.55-6.05)	0.001
Grade≥2 postoperative complication (yes/no)	2.55 (0.78-10.51)	0.349			1.55 (0.83-6.73)	0.257		

Note: *LLN*, lateral lymph node; *TME*, total mesorectal excision; *CEA*, carcinoembryonic antigen; *LLND*, lateral lymph node dissection.

**Table 4:** Univariate and multivariate regression analyses of 62 patients with pathological LLNM.

Variables	DMFS			
	Univariate analysis		Multivariate analysis	
	HR (95%CI)	*P*	HR (95%CI)	*P*
Sex: male/female	1.17 (0.50-2.73)	0.725		
Age at operation (≥65/<65years)	0.55 (0.16-1.87)	0.342		
CEA level (>5/≤5 ng/L)	1.76 (0.73-4.22)	0.205		
Distance from anal verge (>5/≤5 cm)	0.32 (0.11-0.96)	0.041	0.55 (0.17-1.78)	0.318
Histology (Poor, Mucinous or signet/moderate)	3.18 (0.32-12.14)	0.327		
Operative type: laparoscopic/open	0.45 (0.20-1.05)	0.063	0.55 (0.21-1.41)	0.211
Lymphatic invasion (yes/no)	2.83 (0.17-17.15)	0.469		
Perineural invasion (yes/no)	2.02 (0.51-9.35)	0.678		
pT stage (T3-T4/T1-T2)	0.47 (0.14-1.61)	0.228		
pN stage (mesorectal LN)				
N0	-	-		
N1	3.45 (0.43-27.73)	0.275		
N2	5.19 (0.68-39.66)	0.213		
LLNM locations (obturator or internal iliac/other)	2.47 (1.12-5.96)	0.037	2.85 (1.31-4.67)	0.042
LLNM (Bilateral/Unilateral)	2.27 (0.53-9.84)	0.272		

Note: *LLN*, lateral lymph node; *CEA*, carcinoembryonic antigen; *HR*, hazard ratio.

## Abbreviations

LLN: Lateral lymph node
LLNM: Lateral lymph node metastases
LLND: LLN dissection
PSM: propensity score-matched
RFS: recurrence-free survival
LRFS: local recurrence-free survival
DMFS: distant metastasis-free survival
TME: total mesorectal excision
MRI: magnetic resonance imaging
AJCC: American Joint Committee on Cancer
MDT: multidisciplinary team meeting
NCCN: National Comprehensive Cancer Network
EMSO: European Society for Medical Oncology
nCRT: neoadjuvant chemoradiotherapy
ASA: American Society of Anesthesiologists
CEA: carcinoembryonic antigen
HR: hazard ratio
