# Supplementary material for: Distant Metastasis is the Dominant Cause of Treatment Failure after Lateral Lymph Node Dissection in Patients with Lateral Lymph Node Metastasis: Results of the Large Multicenter Lateral Node Study in China
Source: J Cancer. 2023 Oct 2;14(17):3227–37. doi: 10.7150/jca.88009 (PMC10622990; doi:10.7150/jca.88009)
Supplement: Supplementary file 1 — Supplementary table. [file jcav14p3227s1.pdf]

supplementary Table 1. Univariate and multivariate regression analyses of 110 patients with clinical LLNM who underwent TME+LLND

Variables	LRFS				DMFS			
	Univariate analysis		Multivariate analysis		Univariate analysis		Multivariate analysis	
	HR (95%CI)	*P*	HR (95%CI)	*P*	HR (95%CI)	*P*	HR (95%CI)	*P*
Sex: male/female	0.84 (0.35–2.05)	0.413			1.53 (0.58-3.08)	0.832		
Age at operation (≥65/<65years)	0.67 (0.30–4.41)	0.353			0.73 (0.41-2.35)	0.300		
CEA level (>5/≤5 ng/L)	1.90 (0.73–6.05)	0.203			2.05 (0.97-5.84)	0.064	1.62 (0.70–3.05)	0.356
Distance from anal verge (>5/≤5 cm)	0.81 (0.41–2.42)	0.503			0.70 (0.56-4.57)	0.350		
Operative type: laparoscopic/open	1.65 (0.54–4.42)	0.620			1.32 (0.72-9.05)	0.493		
LPND (Bilateral/Unilateral)	0.83 (0.53–2.43)	0.427			0.55 (0.31-1.25)	0.109	0.53 (0.36-3.21)	0.302
Histology (Poor, Mucinous or signet/moderate)	1.46 (0.61–6.13)	0.455			3.52 (1.25-8.31)	0.039	2.93 (0.89–7.03)	0.155
Lymphatic invasion (yes/no)	1.84 (0.39–6.64)	0.532			1.92 (0.33-5.29)	0.783		
Perineural invasion (yes/no)	3.03 (1.40–7.53)	0.046	3.35 (1.64-12.02)	0.038	1.15 (0.68-4.04)	0.342		
pT stage (T3–T4/T1-T2)	3.13 (0.81-8.57)	0.058	2.97 (0.91-10.53)	0.106	0.68 (0.36-4.36)	0.439		
pN stage (mesorectal LN)								
N0	-	-	-	-	-	-	-	-
N1	2.52 (0.96–11.38)	0.073	3.15 (0.85-5.35)	0.287	2.65 (1.13-6.58)	0.045	2.06 (0.80–6.35)	0.140
N2	3.25 (2.31–8.97)	0.012	4.94 (0.91-6.86)	0.105	4.60 (2.13-7.95)	<0.001	3.52 (1.55–8.13)	0.002
Pathological LLNM (yes/no)	2.45 (0.90–5.98)	0.102			3.94 (2.40-10.33)	<0.001	4.01 (1.34-10.01)	<0.001
Grade≥2 postoperative complication (yes/no)	2.32 (0.72–6.53)	0.459			1.46 (0.88-5.90)	0.340		

Note: *LLN*, lateral lymph node; *TME*, total mesorectal excision; *CEA*, carcinoembryonic antigen; *LLND*, lateral lymph node dissection
